# Optimization of low-carbon multi-temperature joint distribution for fresh agricultural products under 3D loading constraints

**DOI:** 10.1371/journal.pone.0353789

**Published:** 2026-07-13

**Authors:** Juping Shao, Fan Gao, Yanan Sun

**Affiliations:** 1 School of Business, Suzhou University of Science and Technology, Suzhou, Jiangsu, China; 2 Anwood Logistics System Co. Ltd, Suzhou, Jiangsu, China; Istinye University: Istinye Universitesi, TÜRKIYE

## Abstract

With the growing demand for fresh agricultural products, improving the efficiency and sustainability of cold-chain distribution has become increasingly important. Multi-temperature joint distribution provides an effective solution for serving products with different temperature requirements, yet its implementation remains challenging due to the need to coordinate vehicle routing, three-dimensional loading, and carbon-emission reduction objectives. To address this issue, this paper develops a low-carbon multi-temperature joint distribution optimization model under three-dimensional loading constraints. A hybrid algorithm integrating genetic algorithm and tabu search is proposed to solve the model efficiently. The proposed approach is validated using real-world data collected from a fresh agricultural products supply chain company. The results show that the multi-temperature joint distribution mode reduces total operating costs by 30.04% and carbon emissions by 30.62% compared with the conventional single-temperature distribution mode. Moreover, the proposed hybrid algorithm achieves faster convergence and better solution quality than the conventional genetic algorithm. These findings demonstrate the effectiveness of integrating three-dimensional loading, multi-temperature distribution, and low-carbon objectives within a unified optimization framework, providing practical support for distribution planning and decision-making in cold-chain logistics.

## 1. Introduction

With the development of technology and economy, people’s quality of life has been continuously improving. Consequently, the demand for fresh agricultural products has been increasing. Fresh agricultural products have distribution requirements of “multiple varieties, small batches, and high quality.” Most existing studies only consider the distribution of goods requiring a single temperature level. In many practical application scenarios, however, transportation often involves various types of goods with different temperature requirements. Accordingly, the Multi-Compartment Vehicle Routing Problem (MCVRP) has been proposed, in which vehicles are equipped with multiple compartments to simultaneously serve goods maintained at different temperatures [1]. In this paper, a novel multi-temperature joint distribution mode is proposed to address the distribution requirements of fresh agricultural products. This mode can effectively improve distribution efficiency and reduce distribution costs.

Meanwhile, the delivery process involves high energy consumption and excessive carbon emissions, which cause environmental impacts [2]. This contradicts the state-advocated concepts of “green logistics” and “low-carbon logistics.” To address high carbon emissions in delivery, this study comprehensively considers carbon emissions throughout the entire process and incorporates low carbon emission reduction as an optimization objective.

In addition, a reasonable loading plan also exerts a significant impact on distribution. In existing studies, the loading process usually only considers vehicle weight capacity constraints, and customer demands are merely simplified to weight attributes. However, in practical transportation scenarios, distribution needs to take into account both routing plans and loading schemes simultaneously, especially when goods have heterogeneous dimensions and loading becomes non-trivial [3]. The integrated research of vehicle routing problems and three-dimensional loading is of great practical significance. Such problems are referred to as the Three-Dimensional Loading Capacitated Vehicle Routing Problem (3L-CVRP) [4]. This paper further incorporates the Multi-Compartment Vehicle Routing Problem (MCVRP) based on this foundation, which better reflects the practical requirements of fresh agricultural product distribution.

Existing studies have made substantial progress in three-dimensional loading vehicle routing, multi-compartment distribution, and low-carbon logistics optimization. However, these research streams have largely evolved independently. In the context of fresh agricultural product distribution, routing decisions, loading feasibility, temperature-control requirements, and carbon emissions are closely intertwined. A routing plan that is efficient from a transportation perspective may become infeasible once loading constraints are considered, while compartment allocation decisions can further affect vehicle utilization and emission performance. This makes it difficult for existing models to fully capture the operational characteristics of fresh cold-chain distribution.

To better reflect practical distribution requirements, this study develops a low-carbon multi-temperature joint distribution optimization model under three-dimensional loading constraints. The proposed framework simultaneously considers routing decisions, loading arrangements, multi-temperature transportation requirements, and carbon-emission costs. Carbon emissions generated by both vehicle transportation and refrigeration operations are incorporated into the optimization process. To solve the resulting problem efficiently, a hybrid algorithm combining Genetic Algorithm (GA) and Tabu Search (TS) is developed, in which tabu search is embedded to strengthen local exploration and reduce the risk of premature convergence.

The remainder of this paper is organized as follows. The Literature Review section reviews the related literature. The Problem Formulation of Low-Carbon Multi-Temperature Joint Distribution under 3D Loading Constraints section describes the low-carbon multi-temperature joint distribution problem with 3D loading constraints and constructs the optimization model. The An Improved Hybrid Algorithm Combining Genetic Algorithm and Tabu Search section designs an improved genetic algorithm to solve the model. The Example Analysis section carries out a case study using real enterprise data and compares different distribution modes and algorithms. Finally, the Conclusions section concludes the work and discusses future research directions.

## 2. Literature review

This study focuses on an integrated optimization problem that combines the Three-Dimensional Loading Capacitated Vehicle Routing Problem (3L-CVRP) with the Multi-Compartment Vehicle Routing Problem (MCVRP). The proposed framework simultaneously considers the loading and distribution characteristics of fresh agricultural products. In addition, environmental considerations are incorporated into the optimization process by accounting for low-carbon logistics objectives, thereby enhancing the practical relevance of the model.

### 2.1. Three-Dimensional Loading Capacitated Vehicle Routing Problems (3L-CVRP)

In the field of logistics optimization, the integration of three-dimensional loading and vehicle routing is commonly referred to as the Three-Dimensional Loading Capacitated Vehicle Routing Problem (3L-CVRP). The objective of 3L-CVRP is to determine a set of delivery routes that satisfies customer demand while minimizing routing costs and ensuring feasible loading arrangements [5]. The problem involves both loading constraints, such as geometric feasibility and non-overlapping requirements, and routing constraints, including flow balance and vehicle operation requirements. Chi and He [6] elaborated on the differences between the pickup and delivery scenarios, clarified the practical constraints specific to pickup operations, such as loading sequence constraint and reloading ban constraint. Küçük and Yildiz [7] added the fixed vehicle cost to the distribution cost. In addition to basic loading constraints, time-window requirements were incorporated to better reflect practical distribution operations. In most previous studies, the no-split constraint was adopted; however, Bortfeldt and Yi [8] employed the split delivery strategy, where a single customer could be served by multiple vehicles. Based on the split delivery constraint, Chen et al. [9] further considered the service time requirements of customers and additionally incorporated the time window constraint. Rajaei et al. [10] examined the split delivery vehicle routing problem, adopting a heterogeneous fleet as the delivery mode and considering the impact of vehicle type constraints on this problem. A related line of research, represented by Meliani et al. [11], investigated the impact of heterogeneous fleets on both loading plans and route design under non-splitting conditions: they investigated the impact of a heterogeneous fleet on vehicle loading plans and distribution route planning under the non-splitting constraint. Beyond traditional single-objective formulations, Phongmoo et al. [12] established a multi-objective optimization model. Under three-dimensional loading constraints, the model took into account both the maximization of profit and the minimization of empty space in the knapsack. Similarly, in the context of multi-objective optimization, Song et al. [13] integrated three objectives—minimizing the total travel distance, minimizing the number of routes, and minimizing the total number of mixed orders within the same pallet—into the total cost function. They further formulated it as a Mixed Integer Linear Programming (MILP) model, which takes the minimization of total cost as its sole objective.

Existing studies have considerably advanced the integration of routing and loading decisions by incorporating practical considerations such as time windows, heterogeneous fleets, split deliveries, and multi-objective optimization. However, most 3L-CVRP models focus on loading feasibility and transportation efficiency, while temperature-controlled distribution requirements receive relatively limited attention. Consequently, their applicability to fresh agricultural product distribution remains constrained.

### 2.2. Multi-Compartment Vehicle Routing Problems (MCVRP)

In addition to loading considerations, the joint distribution of multiple product categories has become an important issue in logistics operations. For instance, in the distribution of fresh agricultural products, items requiring different temperature conditions often need to be delivered simultaneously. As a result, vehicles equipped with multiple compartments have been adopted to simultaneously transport products requiring different temperature conditions. Wang et al. [14] proposed a vehicle compartment partitioning strategy to improve the vehicle loading rate. In the past, retailers utilized single-compartment vehicles (SCVs) for transporting products with specific temperature requirements. However, relying solely on SCVs for products with different temperature requirements often results in higher transportation costs — a challenge that ultimately led to the adoption of multi-compartment vehicles (MCVs) [15]. Hübner and Ostermeier [16] utilized multi-compartment vehicles for distribution, altering both transportation costs and unloading costs during the distribution process. These cost factors were explicitly incorporated into route planning decisions. To better reflect real-world distribution operations, for multi-compartment vehicles, there existed an interdependent relationship between route planning and loading layout [17]. It was necessary to take into account the dimensions of goods and the loading sequence.

Existing MCVRP studies have demonstrated the operational advantages of multi-compartment vehicles in serving products with diverse temperature requirements. However, most studies focus on compartment allocation and routing decisions, while detailed three-dimensional loading constraints are often simplified. Consequently, the feasibility of loading plans may not be fully consistent with practical distribution operations.

### 2.3. Environmentally sustainable vehicle routing problems

As environmental concerns have received increasing attention, carbon-emission considerations have gradually been incorporated into vehicle routing optimization models. Many studies transform carbon emissions into economic costs and integrate them into the optimization process. Jarumaneeroj et al. [18] considered the three objectives of minimizing the total transportation cost, minimizing the total carbon emissions from the use of refrigerated vehicles, and minimizing the quantity loss of fresh agricultural products during long-haul transportation. Their results showed that adjusting vehicle scheduling decisions could reduce carbon emissions and product deterioration, although at the expense of a slight increase in transportation costs. Chen et al. [19] not only included the carbon emission cost in the total cost but also added the penalty cost for freshness degradation, thereby ensuring the freshness of fresh food. Most early studies focused primarily on carbon emissions generated by vehicle fuel consumption during transportation. Chen et al. [20] further considered emissions generated by refrigeration equipment during distribution. Other studies explored how different operational factors influence carbon-emission performance. Pan et al. [21] nvestigated the use of mixed fleets consisting of electric and fuel-powered refrigerated vehicles and examined their environmental impacts. Yao et al. [22] analyzed carbon-emission reduction from the perspective of freshness-preservation investment and found that appropriate preservation measures could contribute to lower emissions. Jia [23] considered multiple sources of environmental impact, including vehicle exhaust emissions, pollution caused by deteriorated products, and transportation noise. These environmental costs were incorporated into the optimization framework to enhance the sustainability performance of distribution operations.

Research on environmentally sustainable vehicle routing has broadened the consideration of carbon emissions, fuel consumption, and product freshness in logistics optimization. Nevertheless, these studies are largely developed within conventional vehicle-routing frameworks. The interactions among three-dimensional loading constraints, multi-temperature distribution requirements, and carbon-emission reduction objectives remain insufficiently explored.

### 2.4. Research gap and positioning of this study

The above review highlights three limitations in the existing literature. First, studies on 3L-CVRP have substantially improved the integration of routing and loading decisions, yet temperature-controlled transportation requirements are rarely incorporated into the modeling framework. Second, research on MCVRP has demonstrated the operational advantages of multi-compartment vehicles, but detailed three-dimensional loading constraints are often simplified or neglected. Third, environmentally sustainable vehicle routing studies have enriched the consideration of carbon emissions and freshness preservation; however, these studies are generally developed within conventional routing frameworks and pay limited attention to the interactions among loading feasibility, compartment allocation, and emission-reduction objectives. As a result, the operational characteristics of fresh agricultural product distribution remain insufficiently represented in existing optimization models.

From a broader perspective, recent optimization studies have increasingly emphasized the integration of multiple operational objectives, system constraints, and advanced solution approaches. For example, recent research has explored the combination of optimization models with machine-learning techniques, robust optimization under uncertainty, sustainable supply chain design, and portfolio-based decision frameworks. These studies demonstrate the value of integrating multiple decision dimensions within a unified optimization framework. However, their primary focus lies in operating room scheduling, project scheduling, supply chain network design, product portfolio optimization, and energy project selection. Comparatively less attention has been devoted to the simultaneous consideration of three-dimensional loading, multi-temperature transportation, and low-carbon distribution in fresh agricultural product logistics.

To address this gap, this study develops an integrated optimization framework for fresh agricultural product distribution that combines three-dimensional loading constraints, multi-temperature joint distribution requirements, and carbon-emission costs. Unlike previous studies that focus on a single operational dimension, the proposed model jointly considers loading feasibility, routing efficiency, temperature-control requirements, and environmental performance. In addition, a hybrid Genetic Algorithm–Tabu Search (GA–TS) approach is developed to efficiently solve the resulting optimization problem. By integrating these dimensions within a unified framework, the proposed study extends existing research on vehicle routing and provides a more realistic decision-support tool for low-carbon cold-chain logistics operations.

## 3. Problem formulation of low-carbon multi-temperature joint distribution under 3d loading constraints

### 3.1. Distribution scenario description

With the increasing emphasis on low-carbon development, cold-chain logistics has paid growing attention to both environmental sustainability and operational efficiency. Consequently, reducing logistics costs and carbon emissions has become an important objective in distribution planning. In addition, vehicle utilization plays a critical role in distribution performance. Therefore, this study incorporates three-dimensional loading considerations into the optimization framework to improve compartment utilization and support more efficient route planning. By increasing loading efficiency, the model can reduce the number of vehicles required while simultaneously lowering transportation costs and carbon emissions.

Fresh agricultural products demand “multi-variety, small-batch and high-quality”. This demand can be satisfied by the multi-temperature joint distribution mode of cold chain logistics. Therefore, a distribution system consisting of one distribution center and multiple customer locations is considered in this study. Each customer requires fresh agricultural products stored under different temperature conditions.

The distribution center offers customers a variety of fresh agricultural products at different temperature settings: ambient, refrigerated, and frozen. Customer orders, comprising diverse fresh products, are consolidated at the distribution center, which then coordinates the dispatch of vehicles. These vehicles, all of the same type, are equipped with three temperature-controlled compartments (ambient, refrigerated, frozen) to accommodate the varying temperature requirements of the products. A single distribution center is established, from which all vehicles depart and return upon completing their delivery tasks. Information regarding delivery distances and customer demands, such as product weight, dimensions, and storage temperature, is available.

Each customer is serviced by one vehicle only, ensuring that the goods transported do not exceed the vehicle’s compartment capacity or weight limit. The proposed model aims to minimize total logistics costs, including vehicle fixed costs (e.g., labor and rental expenses), transportation costs, refrigeration costs, and carbon-emission costs. Carbon emissions generated by both vehicle transportation and refrigeration equipment are converted into monetary costs through a carbon tax mechanism.

Vehicle utilization is closely related to the number of vehicles deployed. Fewer vehicles generally imply higher loading efficiency and lower fixed operating costs. Distribution route planning determines the service sequence of each vehicle and directly affects cargo loading arrangements. Meanwhile, three-dimensional loading optimization improves vehicle space utilization and reduces the need for additional vehicles. As a result, both operating costs and carbon emissions can be effectively reduced.

### 3.2. Model assumptions

The research in this paper is based on the following assumptions:

1. The supplier has a distribution center where the daily needs of each customer point are aggregated in advance. Delivery vehicles depart from the distribution center and return to the distribution center after completing all delivery tasks.
2. The customer’s demand for the day is known and cannot be split, i.e., each customer can only be served by one delivery vehicle.
3. Each delivery package is regarded as a cuboid. This paper does not consider the loading of bagged goods.
4. Goods are not allowed to be placed on their sides or upside down. Rotation is only permitted in the horizontal plane to exchange their length and width.
5. The different temperature-controlled compartments in the delivery vehicle can only contain fresh agricultural products corresponding to their designated temperature requirements.

### 3.3. Notations and Decision Variables

The relevant parameters, symbols and variables of this article are shown in Table 1.

**Table 1:** Related parameters and variables.

Parameters	Define
N	Customer Point Set,N={i,j|0,1,2,3,⋯}
K	Set of Used Distribution Vehicle Numbers,K={k|0,1,2,3,⋯}
$N_{k}$	Set of Customer Points Served by Distribution Vehicle k
s	Set of In-compartment Area Partitions for Distribution Vehicles,s={1,2,3},Numbers represent ambient temperature areas, refrigerated areas, and frozen areas.
u	The number of goods in each temperature at each customer point, u={1,2,3,…}, Numbers represents the sequence of the goods in that temperature layer at the corresponding customer point.
$d_{ij}$	Distance from Customer Pointi to Customer Pointj,i,j∈N
Q	Maximum Load Capacity of Distribution Vehicles
$Q_{\text{0}}$	Curb Weight of Distribution Vehicles
D	Maximum Driving Distance of Distribution Vehicles
$v_{k}$	Driving Speed of Distribution Vehiclek
$q_{i}$	Total Weight of Goods Required at Customer Pointi
$c_{k}$	Fixed Cost of Distribution Vehiclek
$c_{dist}$	Unit Distance Distribution Cost of Delivery Vehicles
$c_{cool}$	Refrigeration Cost per Hour of Delivery Vehicles
ε	Carbon Emissions per Unit of Fuel Consumption
λ	Carbon Emissions per Unit of goods per Unit Distance Traveled by Refrigeration Equipment
ρ	Fuel Consumption per Unit Distance Traveled by Distribution Vehicles
$\rho_{\text{0}}$	Fuel Consumption per Unit Distance Traveled When Vehicles Are Unladen
$\rho^{*}$	Fuel Consumption per Unit Distance Traveled When Vehicles Are Fully Laden
$C_{tax}$	Unit Carbon Tax Price
Variables	
$x_{ik}$	If distribution vehiclek serves customer Pointi,then$x_{ik}=1$,or$x_{ik}=0$
$q_{k}$	Total Weight of Goods Loaded on Distribution Vehicle k
$q_{ij}$	Total Weight of Goods Loaded on the Distribution Vehicle from Customer Pointi to Customer Pointj
$l_{s}$	Y-axis Coordinate of the Upper Right Corner of the Front Side of Compartment Area s in the Distribution Vehicle
$w_{s}$	X-axis Coordinate of the Upper Right Corner of the Front Side of Compartment Area s in the Distribution Vehicle
$h_{s}$	Z-axis Coordinate of the Upper Right Corner of the Front Side of Compartment Area s in the Distribution Vehicle
$x_{ksiu}$	X-axis Coordinate of the Upper Right Corner of the Front Side of the u Good from Customer Pointi in Areas of Distribution Vehiclek
$y_{ksiu}$	Y-axis Coordinate of the Upper Right Corner of the Front Side of the u Good from Customer Pointi in Areas of Distribution Vehiclek
$z_{ksiu}$	Z-axis Coordinate of the Upper Right Corner of the Front Side of the u Good from Customer Pointi in Areas of Distribution Vehiclek
${x^{\text{''}}}_{ksiu}$	X-axis Coordinate of the Lower Left Corner of the Rear Side of the u Good from Customer Pointi in Areas of Distribution Vehiclek
${y^{\text{''}}}_{ksiu}$	Y-axis Coordinate of the Lower Left Corner of the Rear Side of the u Good from Customer Pointi in Areas of Distribution Vehiclek
${z^{\text{''}}}_{ksiu}$	Z-axis Coordinate of the Lower Left Corner of the Rear Side of the u Good from Customer Pointi in Areas of Distribution Vehiclek
$\overset{s}{\underset{i}{m}}$	Quantity of Goods from Customer Pointi Placed in Areas,$u\in \overset{s}{\underset{i}{m}}$
$w_{siu}$	Width of the u Good from Customer Pointi Placed in Areas
$l_{siu}$	Length of the u Good from Customer Pointi Placed in Areas
$z_{siu}$	Height of the u Good from Customer Pointi Placed in Areas
$y_{ijk}$	If distribution vehiclek travels from Customer Pointi to Customer Pointj,then$y_{ijk}=1$,or$y_{ijk}=0$
k	Number of Distribution Vehicles Used

### 3.4. Cost components and objective function analysis

(1) Fixed cost

Fixed cost C1 includes depreciation costs of distribution vehicles, drivers’ salaries, vehicle maintenance expenses, etc. It is calculated as follows:


$$\begin{array}{c} C1=c_{k}\cdot k \end{array}$$ (1)

where $c_{k}$ is the fixed cost per distribution vehicle, and k is the number of distribution vehicles used.

(2) Transportation cost

Transportation cost C2 refers to the fuel consumption cost incurred during the driving of distribution vehicles. It is calculated as follows:


$$\begin{array}{c} C2=\sum_{k\in K}\sum_{i,j\in N}c_{dist}d_{ij}y_{ijk} \end{array}$$ (2)

where $c_{dist}$ is the cost incurred per unit distance traveled by the distribution vehicle.

(3) Refrigeration Cost

As cold-chain vehicles are used for distribution, the operation of refrigeration equipment consumes energy throughout the transportation process, resulting in refrigeration costs. This study only considers the costs incurred by refrigerating the goods in the carriage during transportation and excludes those associated with unloading operations. The refrigeration cost is a function of vehicle travel distance and operating time is calculated as follows:


$$\begin{array}{c} C3=\sum_{k=K}\sum_{i,j\in N}\sum_{s\in S}c_{cool}\frac{d_{ij}}{v_{k}}y_{ijk} \end{array}$$ (3)

where $c_{cool}$ is the cost per unit time of the refrigeration equipment during the transportation process.

(4) Carbon emission cost

Distribution vehicles consume fuel during the delivery process, thereby generating carbon emissions. To incorporate these emissions into the optimization model, a carbon tax mechanism is adopted. The resulting carbon-emission cost depends on the fuel consumption of distribution vehicles is calculated as follows:


$$\begin{array}{c} C4=\sum_{k=1}^{K}\sum_{i=0}^{N}\sum_{j=0}^{N}C_{tax}\cdot \left[d_{ij}\cdot y_{ijk}\cdot \varepsilon \cdot \rho \left(q_{ij}\right)+\lambda \cdot d_{ij}\cdot q_{ij}\right] \end{array}$$ (4)

where $C_{tax}$ is the unit carbon price or carbon tax price.

$d_{ij}\cdot \varepsilon \cdot \rho \left(q_{ij}\right)$is the carbon emissions generated by fuel consumption from transporting goods from customer pointi to customer pointj during the distribution process.

It is known that the unit distance fuel consumption ρ of a vehicle has a linear relationship with its onboard load $q_{k}$, as expressed by the following formula:


$$\begin{array}{c} \rho \left(q_{k}\right)=a(Q_{\text{0}}+q_{k})+b \end{array}$$ (5)

where$Q_{\text{0}}$ is the curb weight of the distribution vehicle.Q is the maximum loading weight of the distribution vehicle.

Let the fuel consumption per unit distance of the distribution vehicle when unloaded be $\rho_{\text{0}}$, and that when fully loaded be $\rho^{*}$. It can be known that:


$$\begin{array}{c} \rho_{\text{0}}=aQ_{\text{0}}+b \end{array}$$ (6)

$$\begin{array}{c} \rho^{*}=a(Q_{\text{0}}+Q)+b \end{array}$$ (7)

By combining Equation (6) and Equation (7), it can obtain: $a=\frac{\rho^{*}-\rho_{\text{0}}}{Q}$, $b=\rho_{\text{0}}-\frac{\rho^{*}-\rho_{\text{0}}}{Q}\cdot Q_{\text{0}}$, substituting into Equation (5), it can obtain: The fuel consumption per unit distance of the distribution vehicle is $\rho \left(q_{k}\right)=\rho_{\text{0}}+\frac{\rho^{*}-\rho_{\text{0}}}{Q}\cdot q_{k}$.

Meanwhile, the refrigeration equipment also consumes fuel and generates carbon emissions when in operation. It is calculated as follows:


$$\begin{array}{c} \lambda \cdot d_{ij}\cdot q_{ij} \end{array}$$ (8)

where λ is carbon emissions per unit of goods per unit distance traveled by refrigeration equipment.

### 3.5. Mathematical formulation of the optimization model

This paper takes minimizing operational costs, minimizing carbon emissions, and maximizing the average vehicle loading rate as objectives. Carbon emissions are converted into monetary costs through a carbon tax mechanism. The loading rate of vehicles can be reflected by the number of vehicles used. Since using fewer vehicles generally implies a higher loading rate, vehicle fixed costs are introduced as a penalty term. In this way, environmental and operational objectives can be evaluated within a unified cost framework, allowing the problem to be formulated as a total-cost minimization model. The total cost is calculated as follows:


$$\begin{array}{c} min\;Z_{\text{1}}=C1+C2+C3+C4 \end{array}$$ (9)

The constraints are as follows:


$$\begin{array}{c} \sum_{k=1}^{K}x_{ik}=1,\forall i\in \left\{1,2,\cdots ,N\right\} \end{array}$$ (10)

$$\begin{array}{c} \sum_{i=1}^{N}y_{ijk}=x_{jk},\forall j\in \left\{1,2,\cdots ,N\right\},\forall k\in K \end{array}$$ (11)

$$\begin{array}{c} \sum_{j=1}^{N}y_{ijk}=x_{ik},\forall i\in \left\{1,2,\cdots ,N\right\},\forall k\in K \end{array}$$ (12)

$$\begin{array}{c} \sum_{i=1}^{N}q_{i}x_{ik}\leq Q,\forall k\in K \end{array}$$ (13)

$$\begin{array}{c} \sum_{j=1}^{N}y_{0jk}=1,\forall k\in K \end{array}$$ (14)

$$\begin{array}{c} \sum_{i=1}^{N}y_{i0k}=1,\forall k\in K \end{array}$$ (15)

$$\begin{array}{c} \sum_{j=1}^{N}y_{jik}=x_{ik}=\sum_{j=1}^{N}y_{ijk},\forall k\in K \end{array}$$ (16)

$$\begin{array}{c} \sum_{i,j\in N}d_{ij}y_{ijk}\leq D,\forall k\in K \end{array}$$ (17)

$$\begin{array}{c} x_{ksiu}<w_{s},y_{ksiu}<l_{s},z_{ksiu}<h_{s} \end{array}$$ (18)

$$\begin{array}{c} \begin{array}{c} {x^{\text{''}}}_{ksiu}\geq x_{ksjv}\vee {x^{\text{''}}}_{ksjv}\geq x_{ksiu}\vee {y^{\text{''}}}_{ksiu}\geq y_{ksjv}\vee {y^{\text{''}}}_{ksjv}\geq y_{ksiu}\\ \vee {z^{\text{''}}}_{ksiu}\geq z_{ksjv}\vee {z^{\text{''}}}_{ksjv}\geq z_{ksiu},\forall k\in K,\forall s\in S,\forall i\in N,u,v\in \overset{s}{\underset{i}{m}} \end{array} \end{array}$$ (19)

$$\begin{array}{c} x_{ksiu}-x_{ksjv}\geq w_{siu}\vee y_{ksiu}-y_{ksjv}\geq l_{siu}\vee z_{ksiu}-z_{ksjv}\geq h_{siu} \end{array}$$ (20)

$$\begin{array}{c} x_{ik}\in \left\{0,1\right\},\forall i\in \left\{1,2,\cdots ,N\right\},\forall k\in K \end{array}$$ (21)

$$\begin{array}{c} y_{ijk}\in \left\{0,1\right\},\forall i,j\in \left\{1,2,\cdots ,N\right\},\forall k\in K \end{array}$$ (22)

Constraint (10) ensures that each customer node is served by only one delivery vehicle. Constraints (11) and (12) ensure that each customer node is visited only once. Constraint (13) specifies the vehicle loading capacity constraint. Constraints (14) and (15) indicate that each vehicle departs from the distribution center and returns to the distribution center after completing its delivery task. Constraint (16) represents the flow conservation constraint, ensuring that a vehicle leaves a customer node after completing service. Constraint (17) defines the maximum travel distance allowed for each vehicle. Constraint (18) specifies the capacity limit of each compartment. Constraint (19) ensures that loaded items do not overlap within the vehicle. Constraint (20) enforces the reverse loading sequence, meaning that goods destined for customers served later are loaded before those destined for customers served earlier. Constraints (21) and (22) define the variable domains.

## 4. An improved hybrid algorithm combining genetic algorithm and Tabu search

### 4.1. Design Rationale of the Hybrid GA-TS Algorithm

Metaheuristic algorithms are widely used to address NP-hard optimization problems. To tackle the problem model, a hybrid approach combining the genetic algorithm and tabu search algorithm is employed. The genetic algorithm is renowned for its robust global search capability, effectively preventing local optimization traps and facilitating the discovery of superior solutions. Nonetheless, it is characterized by slow convergence and limited local search effectiveness. In contrast, the tabu search algorithm excels in local search, mimicking human memory by utilizing a tabu table to restrict revisits to previously explored areas, thereby mitigating local optimization and enhancing solution quality. However, its global search performance is suboptimal. By integrating these algorithms, synergistic benefits are realized, enabling the attainment of improved local optima while averting local optimization traps and maintaining effective global exploration capability.

### 4.2. Hybrid algorithm framework

#### 4.2.1. Genetic algorithm components.

(1) Population Initialization

The delivery route plan for each distribution vehicle, which is the problem object in this paper, is encoded using real numbers. For example, [1,2,4] and [3,5] represent that delivery vehicle 1 delivers in the order of 1-2-4, and delivery vehicle 2 delivers in the order of 3–5. The initial evolutionary generation is set to 1, and initial solutions are generated randomly. Each randomly generated solution is regarded as a feasible candidate solution.

(2) Fitness Calculation

The fitness of each individual in the evaluation population is calculated according to the set objective function.

(3) Selection

Based on the calculated fitness values, the optimal individuals in the population are selected and retained in the new population.

(4) Crossover

Pairs of individuals are selected from the population, and parts of their chromosomes are exchanged at a crossover probability of $P_{c}$ to generate new individuals. For example, performing crossover on the delivery routes [1,2,4] and [3,5] might yield new routes [1,5,4] and [3,2].

(5) Mutation

Individuals in the population are selected, and under the influence of a mutation probability $P_{m}$, one or more genes in the selected individuals are modified, generating new candidate solutions. For example, swapping the positions of 2 and 4 in the route [1,2,4] results in a new route [1,4,2].

(6) Termination Criterion

The maximum evolutionary generation G is set first. The algorithm terminates when the maximum number of generations is reached. The optimal individual of this generation is then output as the optimal solution.

#### 4.2.2. Composition of Tabu search algorithm.

(1) Tabu Object

Tabu objects refer to the individual elements placed in the tabu list. Their purpose is to prevent detours in the search path. This helps the algorithm escape local optima and explore a broader solution space.

(2) Tabu List

The main function of the tabu list is to avoid cycles during the search process, thereby preventing the obtained solution from being trapped in a local optimum. It usually records the movement operations of tabu objects in the solution process (i.e., records tabu individuals) and prohibits the repeated appearance of these tabu objects within a certain period to avoid revisiting previously explored solutions.. After a fixed number of iterations, the tabu list releases these individuals, allowing them to participate in the search process again. It is a circular list and a first-in-first-out queue with a certain length (i.e., tabu tenure).

(3) Tabu Tenure

Tabu tenure refers to the queue length of the tabu list cycle, that is, the maximum number of times a tabu object is prohibited from being selected. When the number of times reaches 0, the tabu object is removed from the tabu list.

### 4.3. Implementation steps of the hybrid algorithm

Initially, solutions are randomly generated and considered as potential solutions, forming an initial distribution scheme. The fitness values of each individual are assessed based on an objective function, which evaluates the total cost of each distribution scheme. The optimal individual, corresponding to the distribution scheme with the lowest cost, is selected by comparing fitness values. A subset of individuals is chosen to create a tabu list, preventing repeated exploration of previously visited solutions. Through genetic algorithm operations such as selection, crossover, and mutation, new individuals are produced and their fitness is continuously evaluated. By integrating the tabu list from the tabu search algorithm, individuals in the tabu list are excluded from selection to avoid revisiting recently explored solutions and to expand the search space, thereby reducing the risk of being trapped in local optima. The process continues until the termination criterion is satisfied, such as reaching the maximum number of iterations.

**Fig 1:**
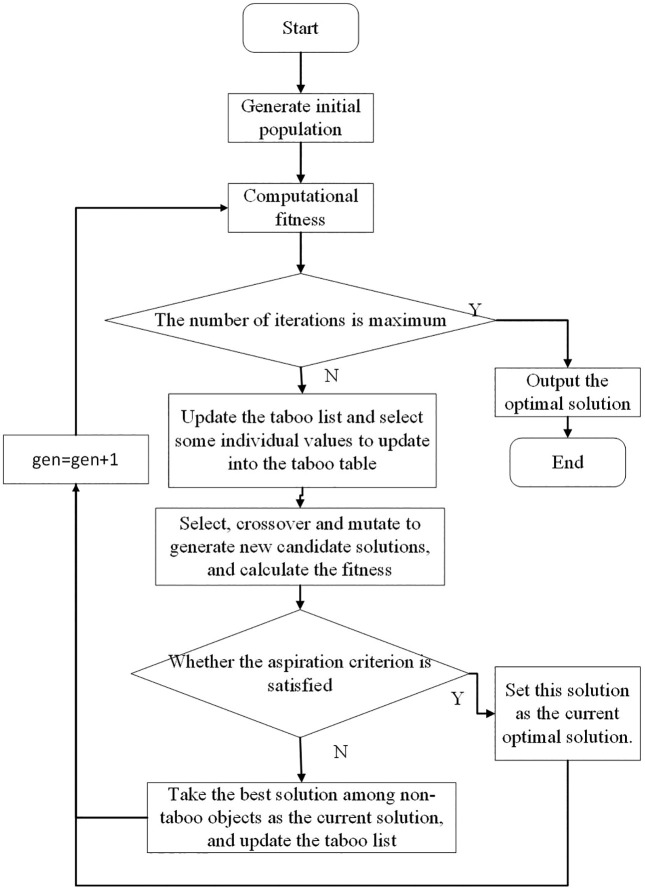
Algorithm flow chart.

The algorithm flow is shown in Fig 1:

## 5. Example analysis

### 5.1. Case background

To verify the designed model and algorithm, this paper uses single-day operational data collected from a chain fresh agricultural products supply chain company in Suzhou through on-site investigations and enterprise interviews. This company adopts a mode where the central warehouse receives customer demands and then arranges deliveries in a unified manner. Data from 10 customer locations were collected, including their distances from the distribution center and detailed daily demand information. The delivery vehicles used are of the same type, with internal dimensions of the carriage being 410 cm * 204 cm * 270 cm. The carriage is equipped with three temperature-controlled compartments, including ambient temperature, refrigerated and frozen.

The values of the model parameters and other relevant parameters used in the case study, which were obtained through field investigations and enterprise interviews, are shown in Table 2.

**Table 2:** Parameter setting.

Parameter	Parameter Value	Parameter	Parameter Value
$v_{k}$	60 km/h	$c_{k}$	500 CNY/vehicle
Q	1500 kg	$c_{dist}$	5 CNY/km
$c_{cool}$	55 CNY/h	λ	0.012 g*kg^-1^*km^-1^
ε	2.66 kg/L	$\rho^{*}$	0.255L/km
$\rho_{\text{0}}$	0.165L/km	$l_{1,2,3}$	2.04 m
$C_{\text{t}ax}$	0.075 CNY/kg	$w_{\text{1}}$	2.10 m
$h_{1,2,3}$	2.70 m	$w_{\text{3}}$	4.10 m
$w_{\text{2}}$	3.10 m		

Table 3 shows the distance matrix between the distribution center and customer points, as well as between customer points. The distances were obtained from navigation software and represent the actual travel distances of delivery vehicles between locations.

**Table 3:** Distance of each distribution point.

Distance（km）	T1	k1	k2	k3	k4	k5	k6	k7	k8	k9	k10
**T1**	0	90	95	91	84	83	77	85	82	84	77
**k1**		0	12	17	8.2	7.3	16	9.9	11	9	20
**k2**			0	2	17	24	10	26	27	21	12
**k3**				0	17	25	9.1	27	24	21	10
**k4**					0	5.3	7.3	7.2	7.2	4.4	15
**k5**						0	15	2.9	3.6	3.9	21
**k6**							0	17	15	12	2.8
**k7**								0	2.3	5.5	22
**k8**									0	3	20
**k9**										0	17
**k10**											0

Table 4 presents the daily demands of the 10 collected customer points. The daily demand of each customer point can be divided into ambient-temperature goods, refrigerated goods and frozen goods according to the transportation temperature of the goods.

The proposed model was implemented in Matlab. By inputting the collected case information, the program generates both the cargo loading plan and the corresponding distribution route plan.

**Table 4:** Demand of each distribution point.

Customer	Ambient temperature goods（box）	Refrigerated goods（box）	Frozen goods（box）
**k1**	18	4	3
**k2**	11	4	2
**k3**	15	5	3
**k4**	12	4	2
**k5**	8	4	3
**k6**	12	4	2
**k7**	17	5	3
**k8**	10	3	2
**k9**	13	5	3
**k10**	14	4	2

### 5.2. Comparison of genetic algorithm and improved hybrid algorithm

Under the condition that the demands of 10 customers were known, the distribution center arranged cold chain vehicles to implement multi-temperature joint distribution. Ambient-temperature goods, refrigerated goods and frozen goods were uniformly loaded and distributed in the same distribution vehicle with different temperature zones. With all other input parameters remaining unchanged, the corresponding distribution route schemes and objective function values were obtained using different solution algorithms.

**Fig 2:**
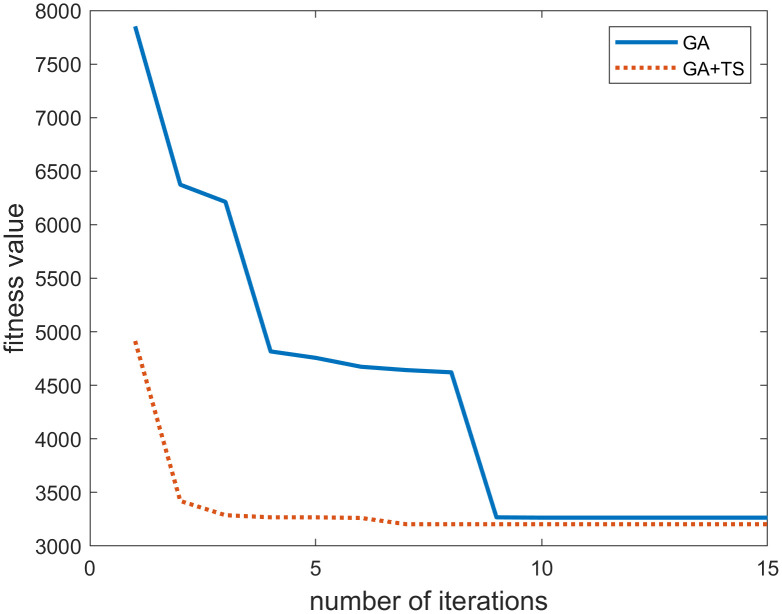
Algorithm Convergence Curve.

Fig 2 shows the optimization process of the objective function, where the horizontal axis represents the number of iterations and the vertical axis represents the individual fitness value, i.e., the objective function. A total of 15 iterations were set in this optimization process.

By comparing the iteration processes of the genetic algorithm and the improved algorithm integrating genetic algorithm and tabu search algorithm, two observations can be made. First, compared with the genetic algorithm, the improved algorithm exhibits a faster convergence rate. Second, the improved algorithm reaches the optimal solution in fewer iterations.

Table 5 presents the distribution route schemes obtained by different algorithms.

**Table 5:** Multi-temperature joint distribution Schemes Obtained by Different Algorithms.

Algorithm	Vehicle number	Route	Load capacity (kg)	Driving distance (km)	Total cost（CNY）	Carbon emissions（kg）
**GA combined with TS**	Vehicle 1	[10,3,2,1,5]	1461.98	191.3	3203.451	206.231
	Vehicle 2	[6,4,9,7,8]	1186.715	178.5		
**GA**	Vehicle 1	[10,6,4,9,8,7]	1462.625	184.8	3264.538	208.2
	Vehicle 2	[5,1,2,3]	1186.07	195.3		

The distribution route scheme obtained by the unimproved genetic algorithm requires 2 vehicles in total, with a total cost of 3,264.538 yuan and a carbon emission of 208.2 kilograms. The scheme derived from the improved algorithm also arranges 2 vehicles, with a total cost of 3,203.451 yuan and carbon emissions of 206.231 kilograms. Compared with the unimproved algorithm, the cost is reduced by 1.87% and the carbon emissions are reduced by 0.94%. These results indicate that the improved algorithm achieves better performance in both cost reduction and emission mitigation.

### 5.3 Comparison of distribution modes

The multi-temperature joint distribution mode is also superior to the single-temperature distribution mode. The multi-temperature joint distribution mode has advantages in improving vehicle compartment utilization and reducing distribution costs. A comparative analysis of the distribution schemes under these two modes demonstrates the practical advantages of multi-temperature joint distribution.

**Fig 3:**
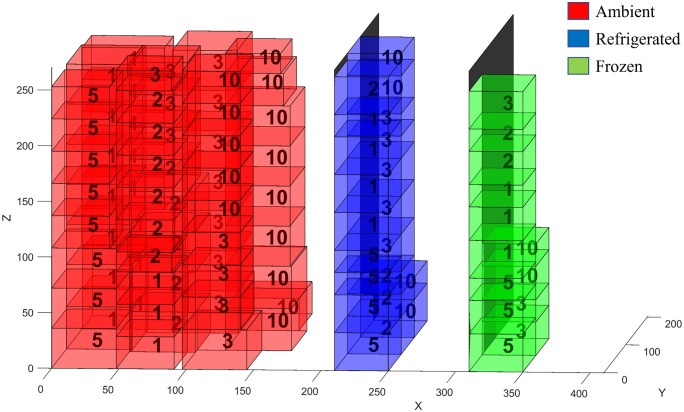
Loading scheme of Vehicle 1 under the Multi-temperature Joint Distribution mode.

**Fig 4:**
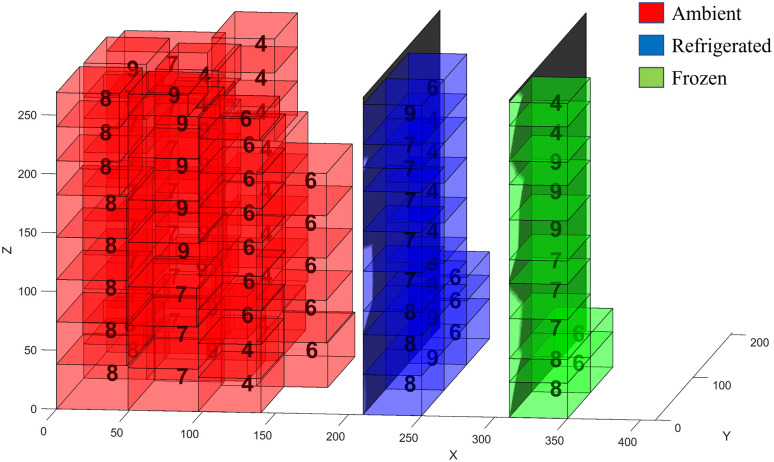
Loading scheme of Vehicle 2 under the Multi-temperature Joint Distribution mode.

Fig 3 and Fig 4 show the cargo loading schemes under the output multi-temperature joint distribution mode.

A total of two loading schemes for Vehicle 1 and Vehicle 2 were output, indicating that the distribution task can be completed with two vehicles. Based on the distribution route schemes in Table 5, the goods for the respective customer points were loaded in the order of delivery, where the numbers on the goods represent the customer points to which they were delivered. As shown in the Fig 3 and Fig 4, the compartment is divided into storage areas for goods of different temperatures. From left to right, they are the ambient-temperature area, refrigerated area, and frozen area, and the respective goods are placed in their corresponding areas.

If the multi-temperature joint distribution is not considered, a single-temperature distribution mode shall be adopted, that is, ambient-temperature goods shall be delivered by ambient-temperature vehicles, and refrigerated goods and frozen goods shall be delivered by cold chain vehicles. The other conditions, such as cargo size, weight, and delivery distance, remain unchanged. A comparison of the resulting loading schemes and distribution routes provides a systematic evaluation of the two distribution modes.

**Fig 5:**
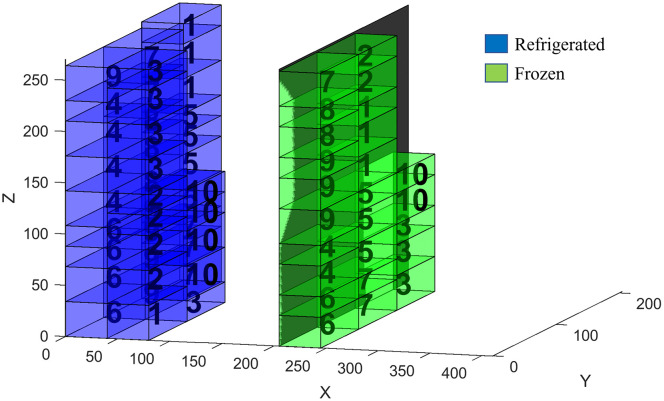
Loading scheme of cold chain vehicle 1 under single temperature distribution mode.

Only the refrigerated and frozen goods required by each customer point were input, and only cold chain vehicles were arranged for distribution. Fig 5 shows the output loading scheme of cold chain vehicles.

A total of one loading scheme for Vehicle 1 was output, indicating that the distribution task can be completed with a single vehicle. Similarly, based on the distribution route scheme shown in Table 6, the goods for the respective customer points were loaded in the order of delivery. Since the goods only include refrigerated and frozen products, the compartment was divided into storage areas for different temperature zones, with the refrigerated area and frozen area arranged from left to right in sequence, and the goods were placed in their corresponding areas accordingly.

**Table 6:** Cold chain vehicle distribution scheme under single temperature distribution mode.

Vehicle number	Route	Load capacity (kg)	Driving distance (km)
**Vehicle 1**	[10,3,2,1,5,7,8,9,4,6]	503.325	205.2

Table 6 presents the distribution route scheme for cold chain vehicles.

A total of one cold chain vehicle is arranged, with the total cost of the scheme being 1,721.642 yuan and the carbon emissions amounting to 100.555 kg.

**Fig 6:**
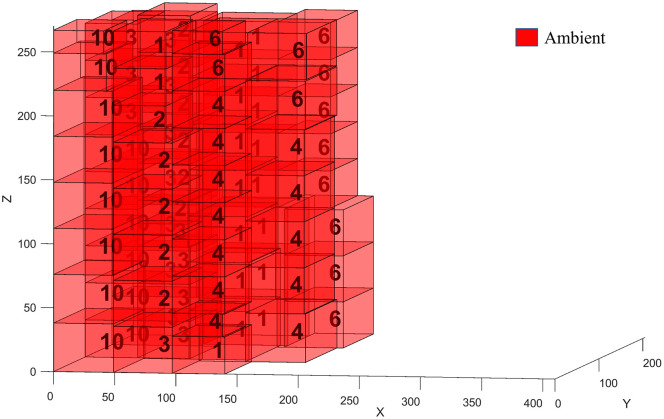
Loading scheme of ambient temperature vehicle 1 under single temperature distribution mode.

**Fig 7:**
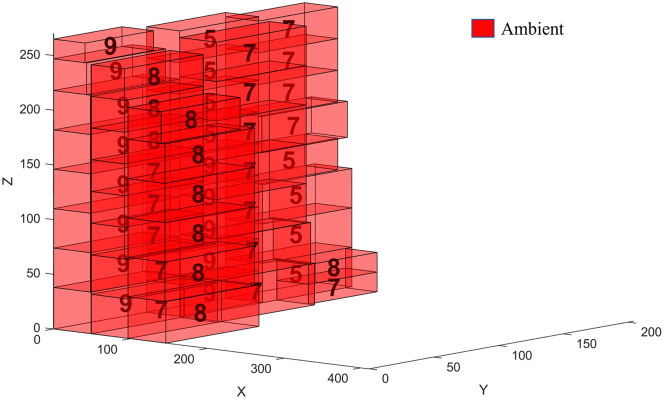
Loading scheme of ambient temperature vehicle 2 under single temperature distribution mode.

When only the ambient-temperature goods required by each customer point were input, ambient-temperature vehicles were arranged for delivery. Fig 6 and Fig 7 show the resulting loading schemes for the ambient-temperature vehicles.

A total of two loading schemes for Vehicle 1 and Vehicle 2 were output, indicating that the distribution task can be completed with two vehicles. Based on the distribution route scheme shown in Table 7, the goods for the respective customer points were loaded in the order of delivery. Since the goods only include ambient-temperature products, they were delivered by ambient-temperature vehicles.

**Table 7:** Ambient temperature vehicle distribution scheme under single temperature distribution mode.

Vehicle number	Route	Load capacity (kg)	Driving distance (km)
**Vehicle 1**	[6,4,1,2,3,10]	1389.53	193.5
**Vehicle 2**	[8,7,5,9]	755.84	175.1

Table 7 presents the distribution route scheme for ambient-temperature vehicles.

This planning scheme arranges a total of 2 ambient-temperature vehicles, with the total cost of the scheme being 2,857.752 yuan and the carbon emissions of 196.691 kg. Because ambient-temperature vehicles do not require refrigeration equipment, refrigeration costs and the associated refrigeration-related carbon emissions are not incurred.

The comparison of the distribution schemes under the multi-temperature joint distribution mode and the single-temperature distribution mode is shown in Table 8.

**Table 8:** Schemes comparison.

mode	Number of vehicles used	Total cost（CNY）	Carbon emissions（kg）
**multi-temperature joint distribution**	2	3203.451	206.231
**single-temperature distribution**	3	4579.394	297.246

In the multi-temperature joint distribution mode, two cold chain vehicles were used to complete the distribution. In the single-temperature distribution mode, two ambient-temperature vehicles and one cold-chain vehicle were required. The multi-temperature joint distribution mode reduces the number of vehicles used and improves vehicle utilization efficiency. In terms of cost, the total operating cost of the multi-temperature distribution mode is lower, representing a reduction of 30.04% compared with the single-temperature distribution mode. In terms of low-carbon emission reduction, the multi-temperature distribution mode achieves a 30.62% reduction in total carbon emissions.

### 5.4. Sensitivity analysis of carbon tax levels

**Fig 8:**
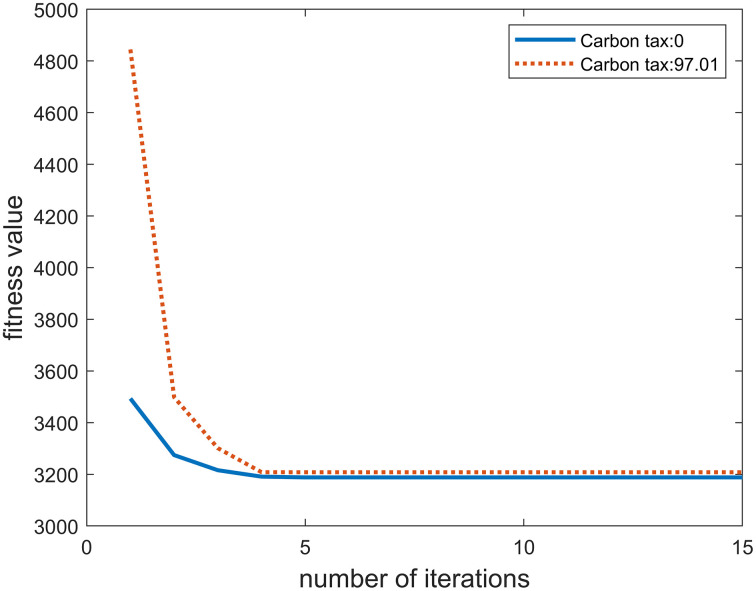
Iteration curves under varying carbon tax prices.

To investigate the model’s sensitivity to carbon tax parameters, we performed numerical experiments under two settings: zero carbon tax and high carbon tax. In 2025, the carbon tax price fluctuated between 50.34 and 97.01 yuan per ton. To investigate the impact of carbon tax price on the model solutions, the carbon tax was set to 0 yuan and 97.01 yuan respectively. The model was solved under otherwise identical conditions, and the iterative process of the solution is shown in the Fig 8.

The obtained distribution plan is presented in the **Table 9**.

**Table 9:** Optimal Solutions with Varying Carbon Tax Prices.

Carbon Tax Price(CNY/kg)	Vehicle number	Route	Load capacity (kg)	Driving distance (km)	Total cost（CNY）	Carbon emissions（kg）
**0**	Vehicle 1	[5,1,2,3,10]	1461.98	191.3	3187.983	208.508
	Vehicle 2	[8,7,9,4,6]	1186.715	178.5		
**0.09701**	Vehicle 1	[10,3,2,1,5]	1462.625	184.8	3207.990	206.231
	Vehicle 2	[6,4,9,7,8]	1186.07	195.3		

Under the zero-carbon-tax scenario, the set of customers assigned to each vehicle remains unchanged, with only the delivery sequence being adjusted. This rearrangement exerts no influence on the total travel distance but leads to an increase in carbon emissions. In the absence of carbon-related costs, the total distribution cost is minimized primarily by shortening delivery distance. Carbon emissions are jointly determined by both travel distance and delivery weight. Even when carbon emission costs are incorporated into the objective function, the optimization model still prefers routes with shorter travel distances. A carbon tax level of 97.01 yuan yields an optimal solution identical to that obtained at 75 yuan. To achieve the lowest possible carbon emissions, the optimization must comprehensively consider both travel distance and delivery sequence rather than distance alone.

Variations in carbon tax price exert a certain impact on the model. Nevertheless, once carbon emission costs are considered, the model will pursue both minimal delivery distance and minimal carbon emissions. As a result, the optimization results tend to be stable.

## 6. Conclusions

This paper studies the optimization problem of multi-temperature joint distribution of fresh agricultural products considering three-dimensional loading constraints from a low-carbon perspective. Transportation costs, refrigeration costs, and carbon-emission costs are incorporated into the optimization framework, with carbon emissions monetized through a carbon tax mechanism. All costs are integrated into the total logistics operation cost, and a cost-minimization model is developed. The proposed model is solved using a hybrid genetic algorithm integrated with tabu search, and its effectiveness is validated through a real-world case study of a chain fresh agricultural products supply chain company in Suzhou. The main findings are summarized as follows:

1. By jointly considering vehicle routing decisions and customer demand characteristics, the proposed model optimizes cargo loading schemes and improves compartment space utilization.
2. The proposed model incorporates vehicle fixed costs as a proxy for vehicle utilization and uses compartment capacity as a key operational constraint. This optimization model fully takes into account the three-dimensional loading constraints and carbon emission costs.
3. A hybrid algorithm that combines the strong global search capability of the genetic algorithm and the strong local search capability of the tabu search algorithm is used to solve the model. The case study results demonstrate that the proposed hybrid algorithm achieves better solution performance than the conventional genetic algorithm.
4. Compared with the single-temperature distribution mode, the multi-temperature joint distribution mode can improve the utilization efficiency of distribution vehicles. It not only effectively reduces costs but also achieves significant improvements in terms of green and low-carbon development, reducing the total carbon emissions during the distribution process.

In general, the case study demonstrates the effectiveness and practical applicability of the proposed model and solution approach. It provides an optimization scheme for the multi-temperature distribution problem considering three-dimensional loading from a low-carbon perspective, and offers solutions for the actual multi-temperature distribution of fresh agricultural products. This paper still has some limitations, and further exploration is expected in future research. (1) This study develops a single-objective optimization model with the total cost as the optimization objective. In practical distribution operations, however, other objectives can also be incorporated, such as minimizing delivery time, maximizing customer satisfaction, and reducing cargo deterioration. Therefore, extending the proposed model to a multi-objective optimization framework represents an important direction for future research. (2) The model in this paper is constructed under deterministic assumptions. In future research, we can extend the proposed model to uncertain environments, such as stochastic demand and uncertain transportation time. The performance and robustness of the algorithm under dynamic and stochastic scenarios will be systematically analyzed, so as to further enhance the practical applicability of the model.

## Supporting information

S1 File Goods dimensions. (XLSX)
